# Partial pictures: what routine health data can and cannot tell us about the quality of maternal and neonatal health services in Tanzania

**DOI:** 10.1093/heapol/czag030

**Published:** 2026-03-05

**Authors:** Jil Molenaar, Amani Kikula, Zamoyoni Julius, Claudia Hanson, Josefien van Olmen, Andrea Barnabas Pembe, Lenka Beňová

**Affiliations:** Family Medicine and Population Health, University of Antwerp, Doornstraat 331, Wilrijk 2610, Belgium; Department of Public Health, Institute of Tropical Medicine Antwerp, Sint-Rochusstraat 43, Antwerp 2000, Belgium; Family Medicine and Population Health, University of Antwerp, Doornstraat 331, Wilrijk 2610, Belgium; Department of Public Health, Institute of Tropical Medicine Antwerp, Sint-Rochusstraat 43, Antwerp 2000, Belgium; Department of Obstetrics and Gynecology, Muhimbili University of Health and Allied Sciences, United Nations Road, P.O. Box 65001, Dar es Salaam, Tanzania; Department of Obstetrics and Gynaecology, Aga Khan University, Urambo Street 344, P.O. Box 38129, Dar es Salaam, Tanzania; Department of Global Public Health, Karolinska Institutet, Tomtebodavägen 18A, Solna 171 65, Sweden; Department of Disease Control, London School of Hygiene & Tropical Medicine, Keppel Street, London WC1E 7HT, United Kingdom; Family Medicine and Population Health, University of Antwerp, Doornstraat 331, Wilrijk 2610, Belgium; Department of Obstetrics and Gynecology, Muhimbili University of Health and Allied Sciences, United Nations Road, P.O. Box 65001, Dar es Salaam, Tanzania; Department of Public Health, Institute of Tropical Medicine Antwerp, Sint-Rochusstraat 43, Antwerp 2000, Belgium

**Keywords:** quality of care, maternal health, neonatal health, routine health information systems, Tanzania, qualitative research, boundary object theory

## Abstract

Measurement of contact coverage of maternal and newborn health services may overestimate the benefits of the care that is provided, because the quality of care is not captured. It is therefore important to better understand the opportunities and challenges of capturing elements of care quality in routine health information systems (RHIS). This study explored healthcare workers’ (HCWs), health sector managers’ and policymakers’ perspectives on the value and limits of routine health data to understand the quality of maternal and neonatal health services in Tanzania. We conducted qualitative research during two periods to capture perspectives across facility, district, regional and national levels. In Mtwara region in 2023, we conducted ethnographic observations at two hospital labour wards and 29 in-depth interviews with healthcare workers, hospital leaders, and district/regional managers. In 2025, we carried out 17 additional interviews with regional managers in Mtwara and with key national-level stakeholders. Our findings demonstrate that Tanzania’s RHIS provides a valuable but partial picture of quality of care for maternal and neonatal health services. Care processes—including both provision and experience of care—are captured only to a limited extent. Using boundary object theory, we highlight how the same health information must serve diverse stakeholder needs. While there are opportunities to integrate more quality-of-care indicators, standardized RHIS cannot be expected to comprehensively capture the multifaceted nature of care quality. For quality measurement to support meaningful local health service improvement, a flexible, bottom-up approach is essential. However, the current emphasis on top-down oversight acts as a barrier for local-level data use. Relevant and feasible measurement of the quality of facility-based care requires a fundamental shift in current RHIS priorities—from systems that extract data for those ‘up there’ to platforms that also create value for those who provide care.

Key messages

- Routine health data have the potential to offer fine-grained insight into the quality of care provided to women and newborns.
- Rigid standardization of quality-of-care measurement is untenable and will fail to accommodate local quality improvement needs in settings like Tanzania, requiring more flexible and adaptable approaches designed from the bottom-up.
- RHIS work best when they create value for those who generate the data, not just those who consume it for oversight purposes, requiring a reorientation to prioritize care over control across health system levels.
- Individual electronic health records combined with patient feedback mechanisms hold promise for improving Tanzania's RHIS, but transforming the system requires fundamental shifts in who defines measurement priorities and whose knowledge counts in system design.

## Introduction

Access to maternal and neonatal health services is crucial to improve health outcomes. Antenatal care (ANC) provides a critical opportunity for early identification of potential risks, while facility-based childbirth care ensures women and neonates can be attended by skilled personnel with referral system linkages in case of complications (Parkhurst et al. 2006, WHO 2016a). Service contact coverage indicators represent the percentage of people who reach a health service among those who need it, and these measures have traditionally been key approaches to evaluating progress towards global goals (Marsh et al. 2020). However, service contact coverage does not account for the quality of service delivery and may therefore overestimate the benefits of the care that is provided (Gabrysch et al. 2019, Strong et al. 2024). This limitation is evident in contexts where maternal and neonatal deaths remain persistently high, even as many more women access antenatal, intrapartum and postpartum care.

Quality of care is a multidimensional concept. Donabedian's (1988) seminal framework defined quality of care through three interconnected elements: the structural context of care delivery, the processes involved in providing care, and the outcomes these generate in the population served. The WHO Quality of Care Framework for Maternal and Newborn Health incorporated these three dimensions and notably placed an equal emphasis both on how care is delivered and how patients experience that care (WHO 2016b). This dual focus thus includes not just the quality of clinical provision of care, but also the need for that care to be responsive to patients’ needs and preferences.

The shift from an almost exclusive focus on measuring coverage and outcomes towards greater consideration of processes and experiences also raises a new set of measurement challenges. The more complex a construct is, the more difficult and weighty the decisions about how to quantify it are (Rottenburg and Merry 2015). Which dimensions of quality of care should be incorporated in measurement, and whose perspectives are prioritized? A qualitative study in Malawi found that perceptions of what constitutes good quality of care differed substantially between maternity care providers and women who had recently given birth (Mgawadere et al. 2019). It has also been noted that receiving evidence-based care from trained providers does not necessarily go hand in hand with a positive experience of care, and vice versa. For example, research in Kenya and Namibia using data from the Service Provision Assessment (SPA) survey found that patient satisfaction with maternal care services was not consistently associated with clinical measures of quality, suggesting that clinical quality of care and patient experience may deviate considerably (Diamond-Smith et al. 2016). Evidently, different measures capture different perspectives and hereby give different types of information about quality of care.

Routine health information systems (RHIS) gather data directly at the health facility or community level and can offer fine-grained insights for management and planning across health system levels (WHO 2023). Sources of RHIS data include facility-based service records, individual record systems, and resource record systems (e.g. for financial management, human resources, or logistics management) (MEASURE Evaluation 2016). The World Health Organization (WHO) has recommended that ideally, RHIS should comprise interoperable data systems that enable health data to be shared and used for multiple purposes and by different health system stakeholders (WHO 2023). When well-implemented, RHIS provide near real-time, continuously available information. This makes them better suited to help inform iterative quality improvement efforts than infrequent household surveys and health facility assessments (Strong et al. 2024). Routine health data typically provide outcome indicators (e.g. mortality) as well as input and process indicators covering resource and infrastructural availability, service delivery, and clinical processes. By combining routine indicators of care coverage with indicators reflecting dimensions of quality of care, quality-adjusted coverage can be assessed, also referred to as effective coverage (Marsh et al. 2020). The Effective Coverage Think Tank Group (ECTTG) defined effective coverage for routine antenatal, childbirth, and postnatal care as the proportion of women who receive ‘timely, appropriate, responsive, and respectful care and treatment’ (Marsh et al. 2020). Frequent monitoring of selected quality-of-care indicators can help HCWs and managers to continuously adapt priorities and strategies as they work together to improve care (WHO 2025).

However, use of quality-of-care indicators from RHIS is far from standard practice. Issues related to data completeness, timeliness, and accuracy continue to hamper RHIS (Nyamtema 2010, Maïga et al. 2019). Given these ongoing challenges, important questions remain about what we can reasonably expect from RHIS in terms of quality-of-care measurement and where our ambitions should lie. In recent years, there have been repeated calls for more research across diverse country contexts to better understand opportunities and complexities in quality-of-care measurement for maternal and neonatal health services and interventions using RHIS (Marsh et al. 2020, Strong et al. 2024).

This study responds to these calls in the Tanzanian context. Tanzania’s RHIS evolved from being fully paper-based in the 1990s, to a hybrid paper-electronic system with nation-wide use of District Health Information Software 2 (DHIS2) for monthly digital summary information entry at the district level by 2014 (Darcy et al. 2017). In 2015–2016, Tanzania established data use and quality frameworks, and the country’s 2021–2026 Health Sector Strategic Plan V (HSSP V) declared that RHIS data should drive evidence-based management and planning across all health sector levels (Darcy et al. 2017, HSSP 2021). Despite high data completeness in Tanzania’s DHIS2 reporting, data utilization remains limited (Mboera et al. 2021). Data quality issues also persist, including implausibly low reported rates of obstetric complications (Shabani et al. 2023). These challenges are partly linked to understaffed clinical environments with high workloads, creating difficult conditions for documentation. HCWs’ fear of blame and punishment for poor outcomes may also impact data capture and use. Data manipulation, often referred to as ‘cooking’ of maternal and neonatal health data, has been observed across diverse settings, including in Tanzania (Melberg et al. 2018, Das et al. 2022, Unkels et al. 2023, Strong 2024, Molenaar et al. 2025). Such creative work-arounds are also linked to a limited sense of data ownership among HCWs (Molenaar et al. 2024b). Indeed, health information systems in high-mortality settings like Tanzania were primarily designed to fulfil higher-level reporting requirements rather than support facility-based quality improvement efforts (Agweyu et al. 2023). This has created a disconnection between system design and frontline needs.

This study aimed to explore the perspectives of HCWs, health sector managers, and policymakers on the value and limits of routine health data to understand the quality of maternal and neonatal health services in Tanzania. We sought to identify what stakeholders perceive as opportunities and challenges within the current system, as well as their priorities for future development. By combining views across health system levels—including people with diverse information needs facing different working environments and constraints—we aimed to provide a comprehensive analysis that can inform future improvements to Tanzania’s RHIS.

## Methods

### Study design

Qualitative data were collected in Tanzania during two time periods. The first data collection phase in Mtwara region (October–December 2023) included ethnographic observations at two hospital labour wards and 29 in-depth interviews with HCWs, hospital leaders, and district- and regional-level managers. During the second phase (January–March 2025), we returned to Mtwara for follow-up conversations. We also conducted 17 additional interviews, including with regional-level managers in Mtwara, as well as with key national-level stakeholders from relevant ministries, UN offices, and development partners in Dodoma and Dar es Salaam.

### Theoretical framework

**Figure 1:**
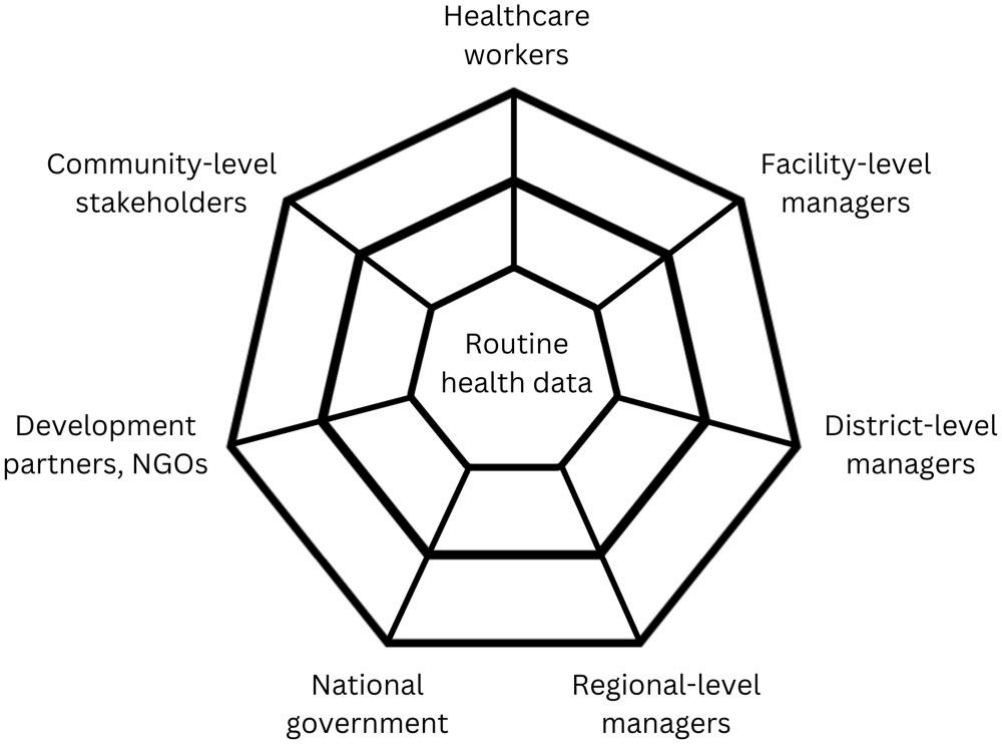
Routine health data as a boundary object.

We conceptualize routine health data as socially significant, as these data both reflect and impact the way people interact (Unkels et al. 2023, Molenaar et al. 2025). We draw on the concept of a ‘boundary object’ (Star and Griesemer 1989) to understand how Tanzania’s RHIS functions across different stakeholder groups. Boundary objects are information or tools that are flexible enough to be used and interpreted differently by various groups. They allow for collaborative work across contexts, while still being flexible enough to adapt to local needs and constraints. Boundary objects act as bridges between different groups of people, allowing them to share information, negotiate meaning, and work together effectively. We can think of the RHIS like a web that connects stakeholders in the health system (Fig. 1). Various actors—healthcare workers, facility managers, district officials, policymakers, and others—are positioned around this web, with the RHIS providing the connecting strands that allow them to ‘meet in the middle’ through shared information. We use this framework to explore how Tanzania’s RHIS currently functions as a boundary object for measuring quality of care, examining both where it succeeds in bridging stakeholder needs and where these connections break down, and why.

### Study setting

Tanzania's health system is hierarchically structured, ranging from village health posts up to national referral hospitals. The Ministry of Health (MoH) regulates healthcare facilities across this spectrum, while the Prime Minister's Office—Regional Administration and Local Government (PMO-RALG) coordinates regional and local government authorities in the implementation of health policies and management at the primary healthcare level. The two most recent Tanzania Demographic and Health Surveys (TDHS) conducted in 2015–16 and 2022 reported a dramatic reduction in the country’s pregnancy-related mortality ratio per 100 000 live births from 556 to 104 per 100 000 live births (DHS 2016, 2023).

Our subnational data collection took place in Mtwara, a southern region of 1.6 million people characterized by subsistence farming. Mtwara has a relatively low fertility rate (3.3 children per woman) and a high percentage of births taking place in health facilities (97%) compared with the national averages of 4.8 and 81%, respectively (NBS 2022, DHS 2023). Observations and interviews with HCWs were conducted at two hospitals in the region. Hospital A is a well-equipped faith-based facility with around 2000 annual deliveries, while Hospital B is a public rural district hospital with 3000 annual deliveries that faces more severe infrastructural challenges, including intermittent running water and electricity, and frequent supply shortages. Both hospitals rely primarily on certificate- or diploma-level nurse-midwives for labour ward care. Staffing levels differ, however, with Hospital A’s labour ward typically having 2–3 per shift compared with Hospital B's 1–2 nurse-midwives covering both antenatal and labour wards.

### Data collection

Data collection in 2023 focused on exploring experiences with routine maternal and neonatal health data at facility, district and regional level in Mtwara. This qualitative study combined focused ethnographic observations with 29 semi-structured interviews conducted between October–December 2023 (Table 1). The first author (J.M.) spent 3 weeks in each of two hospital labour wards (6 weeks total), conducting 230 hours of unstructured observations and conversations across day, evening, night, and weekend shifts. Observations focused on documentation and data reporting practices, including daily routines, division of responsibilities, overlaps between documentation types, and HCWs’ social interactions and communication around documentation tasks. J.M. kept detailed fieldnotes throughout, which were analysed alongside and triangulated with interview transcripts. Interview participants included labour ward HCWs (*n* = 18) and individuals with managerial or data-specific roles at hospital, district, and regional levels (*n* = 11). All available labour ward staff at the two hospitals were invited for an interview, and the other participants were purposively selected to include diverse perspectives on maternal and neonatal health data management, including hospital management, hospital HMIS focal persons, members of the Council Health Management Team (CHMT) from both districts, and members of the Regional Health Management Team (RHMT). More details of the qualitative data collection in 2023 have been provided elsewhere (Molenaar et al. 2024b, 2025).

**Table 1.** Participant characteristics of interviews carried out in 2023 (*n* = 29).

Current professional role	Gender		Total years of work experience			
	Man	Woman	1–9	10–19	20–29	30+
Labour ward HCWs *n* = 18 Nurse-midwives *n* = 14 Maternity-in-charges (nurse-midwives) *n* = 2 Medical doctor or doctor in training *n* = 2	9	9	16	1	0	1
Managers *n* = 11 Hospital-level managers *n* = 3 District-level managers (CHMT) *n* = 5 Regional-level managers (RHMT) *n* = 3^a^	5	6	3	5	3	0

^a^Out of the three RHMT members interviewed in 2023, two were interviewed again in 2025 for follow-up.

The second data collection period took place over the course of 10 weeks in January 2025 to March 2025. J.M. and Z.J. returned to Mtwara to share findings from the 2023 work and to have follow-up conversations with interlocutors. We organized separate meetings with RHMT members (including the Regional Medical Officer, Regional Reproductive and Child Health Coordinator, and HMIS focal person), CHMT members from both districts (District Medical Officer, District Reproductive and Child Health Coordinator, and District HMIS focal person), hospital management and HMIS focal persons from both hospitals and labour ward HCWs at both facilities. We explicitly prompted discussion on quality-of-care measurement, building on findings from 2023. J.M. took extensive pseudonymized notes to capture key points, which she subsequently discussed with Z.J. and expanded based on his recollections. In addition, we conducted three (recorded) follow-up interviews with RHMT members in Mtwara.

For the national-level interviews in 2025, we purposefully sampled key informants with in-depth knowledge of and experience with maternal and neonatal health data systems in Tanzania. J.M. and A.K. interviewed 14 national-level stakeholders, of whom four were current government officials at the MoH or at PMO-RALG. Additional six interviewees had previously held similar governmental positions and/or were currently supporting relevant governmental activities and programmes with external funding (e.g. Global Financing Facility). We also interviewed key stakeholders at relevant UN country offices (WHO and UNICEF), and people involved with Tanzania’s Health Information Systems Programme (HISP), which supports DHIS2 implementation, and with the Tanzanian Demographic and Health Survey (TDHS). We relied heavily on snowball sampling, inviting additional interviewees based on suggestions from initial participants. Five of these interviews took place in Tanzania’s administrative capital Dodoma, five in Dar es Salaam, and four were conducted online (Table 2).

**Table 2.** Participant characteristics of interviews carried out in 2025 (*n* = 17).

Current professional role	Gender		Total years of work experience		
	Man	Woman	10–19	20–29	30+
Regional-level managers (RHMT) *n* = 3^a^	2	1	1	2	0
National-level stakeholders *n* = 14	8	6	5	6	3

^a^Out of the three RHMT members interviewed in 2023, two were interviewed again in 2025 for follow-up.

All 2023 interviews were conducted in Swahili. In 2025, five interviews were conducted mostly in Swahili and twelve mostly in English, with many including a mix of both languages as respondents could choose their preferred language. Different topic guides were used for each fieldwork period (S1), although all included a focus on quality-of-care measurement. In the 2023 data collection in Mtwara, we focused mainly on (1) general experiences with routine maternal and neonatal health data and (2) specific experiences with a perinatal eRegistry. In 2025 interviews, we focused on exploring key stakeholders’ views of how Tanzania’s data systems for maternal and neonatal health have evolved over time, how the information is used for decision-making, and quality-of-care measurement. Interviews were audio-recorded and transcribed verbatim in their original language, with translation from Swahili to English occurring only after analysis for quotes considered for inclusion in the manuscript. Additional reflections on language, researcher positionality and power relations can be found in our reflexivity statement.

### Analysis

Reflexive thematic analysis was conducted in several phases, following Braun and Clarke's methodology (2021) and using NVivo. Initial analysis began during data collection through regular field note reviews, with research team debrief discussions held during and after data collection for collaborative interpretation. Following completion of 2023 interviews, a subset of transcripts was double-coded by J.M. and A.K. to develop a shared coding tree, which J.M. then used to code all 2023 interviews. This coding tree included a focus on quality of care measurement but also covered a wide range of other themes relating to routine maternal and neonatal health data, reflecting the broader scope of the 2023 data collection. For 2025 data, J.M. again coded all transcripts while A.K. coded three transcripts, with the coding tree refined through discussion between both researchers. This iterative approach to coding reflected both the evolving focus of data collection and our developing theoretical framework. The 2025 coding tree had a more comprehensive focus on quality of care measurement, as insights from the 2023 analysis had allowed for more targeted exploration of this topic in 2025. For the write-up of this paper, we identified all codes related to quality of care measurement from both coding trees and reorganized them using our boundary object theory framework, merging codes where they captured similar findings.

## Results

We present our results in four sections. We begin by examining where the system works well—highlighting how routine health data successfully bridge different stakeholder perspectives and discussing what aspects of maternal and neonatal care quality can be understood through these data. We then show where the RHIS provides only a partial picture of care quality and identify key gaps. Third, to understand why these limitations exist, we zoom out and analyse deeper systemic constraints that shape how health information is captured and used. We examine how historical legacies and contemporary power dynamics shape prioritization of top-down oversight and accountability over context-specific information needs. Finally, we look ahead to emerging initiatives that might help reimagine Tanzania's RHIS in ways that better support understanding and improving quality of care.

### Landscape picture: Tanzania’s RHIS and what it can tell us about quality of care

Tanzania’s RHIS consists of a range of different systems and platforms, referred to by a national-level stakeholder (NS) as a ‘heterogeneous ecosystem’ (IV34-NS) that combines both paper-based and electronic forms of documentation. An overview of the main systems used for routine maternal and neonatal health data is provided in Table 3. The RHIS landscape is characterized by considerable fragmentation and parallel reporting requirements. HCWs frequently document the same information across multiple tools and platforms—e.g. recording a woman’s details in paper-based clinical files, copying them into paper MTUHA registers as well as into the hospital’s local electronic health management system (eHMS), and later entering aggregated data into the electronic platform of DHIS2. For the scope of this analysis, we focus primarily on the systems most frequently cited by study participants: the paper-based MTUHA, the digital DHIS2 platform, along with GoTHOMIS.

**Table 3.** Main systems and tools used for collecting and reporting maternal and neonatal health data in Tanzanian health facilities, adapted from Molenaar et al. (2025).

System or tool for collecting and reporting data	Format and function	Mandated by
MTUHA registers	The paper-based component of Tanzania’s HMIS (*Mfumo wa Taarifa za Uendeshaji wa Huduma za Afya*). HCWs record information on the health status of the woman and her baby and the health services provided to them in large, standardised register books (e.g. Book 12: Labour and delivery register; Book 13: Postnatal register). There are also registers on medicines, supplies and human resources. These data are then aggregated using tally sheets and reported through monthly summary forms.	Ministry of Health
DHIS2	Monthly summary forms from MTUHA are entered into the District Health Information Software 2 (DHIS2). This entry of aggregated data about women and babies is done at the district health office, or at the facility level in referral hospitals (by HMIS focal persons).	Ministry of Health
GoTHOMIS	The Government of Tanzania Hospital Management Information System, an electronic health record system that allows for the sharing of patient data within and across primary healthcare facilities. Centralized GoTHOMIS was rolled out starting late 2023.	Prime Minister's Office Regional Administration and Local Government
ANC card (RCH-4)	During antenatal care (ANC), women are given a printed patient-held card, which they are supposed to bring with them every time they come to the hospital. This individual record of a woman’s medical and obstetric history is filled out by HCWs during ANC visits.	Ministry of Health
Clinical patient file	Various paper-based tools constitute a woman’s clinical patient file, including her admission form, observation charts, clinical notes and the partograph for labour monitoring, all filled out by HCWs.	Ministry of Health and/or hospital management
Electronic health information system	Electronic health record (EHR) systems used mostly at higher-level facilities, e.g. Afya eHMS and Care2X. Some EHRs focus primarily on clinical information, while others have more advanced features and modules that support integrating various data types (e.g. billing and financial records, appointment schedules, lab results).	Hospital management
Informal documentation	A variety of informal documentation at the labour wards include non-standardised staff attendance registers, surgery record books, doctors’ rounds notebooks, shift hand-over notes, stillbirth registers, referral books, and drug-specific registers, usually in the form of hand-drawn tables or lists within notebooks completed by HCWs.	Hospital management or HCWs themselves

By design, routine maternal and neonatal health data are intended to have different uses for different users. A national-level stakeholder remarked that ‘the purpose of looking at data depends on who you are, and what your responsibility is’ (IV44-NS). While many HCWs struggled to articulate specific examples of how they themselves used data in their day-to-day working reality, they readily pointed out the importance of data use at the health facility-level. A district-level stakeholder (DS) who also still practiced clinically noted:

‘If we are collecting it well and it's filled out properly, it helps us first to see in our hospital how many clients we are serving, but we can also measure ourselves and see what problems our mothers are encountering. We can measure ourselves to see how many of those who have problems came out safely from our hands, and how many lost their babies maybe, or even died themselves.’ (IV15-DS)

In line with this quote, many HCWs discussed the usefulness of data for administrative purposes such as tracking of patient flows and to identify areas for improvement in their health facility. At the district-level, the Council Health Management Teams (CHMTs) reported using data to plan, budget and manage financial resources. Data are also ‘increasingly [used] to guide supportive supervision’ (IV44-NS) at both the district and regional levels. Finally, at the national level, data are used not just for monitoring of service delivery and resource allocation, but also to inform policy and decision-making: ‘Because it's here where you set policies, you design programs to respond to specific challenges’ (IV40-NS). Routine maternal and neonatal health data should thus create value across health system levels: ‘When data is good it helps all parts, from the service provider down here, up to the government, donors, because they can plan according to the information which we provide’ (IV29-HCW).

Interviewees discussed how RHIS data can provide insights into various dimensions of quality of care. Firstly, they can tell us about a facility’s readiness to care for women and newborns. Routinely collected information about facility characteristics can ‘give a picture of how ready the facility is to provide MNCH care’, including ‘availability of equipment and supplies’ (IV45-NS), as well as whether HCWs have received ‘basic training on the services that they are providing’ (IV42-NS). Routine health data also provide information about the coverage of specific services or interventions. For example, you can ‘take the number of clients looking for a certain service’ such as all women attending ANC, and then take the number of women who received the entire package of services they should have received at their ANC contact as the numerator to get a ‘proxy quality of care indicator’ (IV42-NS). In the case of ANC, this can also link to timing, as DHIS2 tracks the number of women who attend ANC before 12 weeks of gestational age. Attendance within the first 12 weeks allows potential risk factors to be identified early and appropriate preventative care to be provided: ‘because when she arrives, even if she doesn't have anaemia yet, right away she starts to be given that iron supplement, […] we start right away’ (IV39-NS). This example illustrates how the distinction between coverage and quality measures is not always clear-cut, as intervention coverage indicators can provide relevant insights into quality of care.

Routine health data can thus provide important information that is relevant for individual-level clinical intervention and also for facility or even population-level monitoring and management. While population denominators can be challenging to determine, several district- and regional-level stakeholders discussed how facility-based trends often trigger broader community-level interventions, such as targeted health education, that extend beyond individual patient care. This exemplifies how the RHIS can serve as a boundary object—a common framework that maintains meaning across diverse stakeholder perspectives while facilitating coordination between them. Information that is relevant to HCWs at the point of care because it triggers specific clinical actions, once aggregated can be used to understand coverage and service access for district, regional, and national-level managers and policymakers, informing planning and resource allocation. Particularly when indicators are also meaningful from a clinical perspective, they can help create a shared vocabulary that holds value both for facility-level quality improvement and for understanding broader systemic quality issues.

### Partial picture: the limits of RHIS to understand quality of care

Interviewees generally agreed that currently available routine health data provide only a limited view of quality of care. A national-level stakeholder felt that truly understanding the quality of care that women and babies receive ‘will be difficult using routine data’, because you can get ‘a partial picture, but not a real picture’ (IV45-NS). Interviewees across health system levels mentioned patient experience as the dimension of quality of care that is not present in the RHIS. A nurse-midwife noted how aspects of care which are important to patients, such as clear and respectful communication, ‘in the data there, they don’t exist’ (IV18-HCW). The absence of clients’ perceptions leaves a gap in our understanding of how services are being provided:

‘If they [women] would talk about issues like ‘when I entered the delivery room, those things were not handled well,’ then we know for sure that we have a gap here with the services we provide.’ (IV15-DS/HCW)

A national-level stakeholder concluded that ‘we have really done very well on the provision of care side—we have a lot of data’. However, the experience of care dimension is ‘an area to explore and do more’ (IV40-NS).

Interviewees also discussed the difficulty of tracking care pathways and continuity of care using routine health data. While DHIS2 captures obstetric risk factors identified during ANC, for example, it cannot track what happens to patients afterwards. The data show facility attendance or diagnoses but do not confirm whether appropriate, high-quality care followed:

‘There’s an indicator that tells you: this mother has severe anaemia or mild anaemia. […] You identify her, yes, everyone will say the assumption is she was treated, but at the end of the day, where is she? She's not there!’ (IV39-NS)

The sequence and timing of care—critical dimensions of quality of care—remain largely uncaptured in current systems:

‘Was the quality good in terms of, was this the right treatment? What is the turnaround time, and all this? These are key elements in terms of the quality of care.’ (IV34-NS)

This concern extends to neonatal care as well. Another national-level interviewee illustrated how crucial timing is when treating newborns requiring Continuous Positive Airway Pressure (CPAP), a respiratory support system:

‘Even getting the proper treatment at the right time. Because for example, the baby needs CPAP, with all the indications that this baby has difficulty breathing […]. But now the timing from when it is determined until the baby receives that intervention… It's also challenging.’ (IV46-NS)

Interviewees emphasized that standardized RHIS, while valuable for monitoring, planning and learning, cannot realistically provide all the granular information needed for quality improvement efforts at the facility level. Respondents across health system levels pointed out that Tanzania’s current main systems—MTUHA/DHIS2 and GoTHOMIS—are too rigid to capture facility-specific quality challenges, requiring additional targeted data capture. A national-level interviewee emphasized actionable quality measurement must be facility-driven:

‘It needs a process designed in a facility. It is not something that is available and you can enter in MTUHA, it has to be something extra that you do there.’ (IV40-NS)

This perspective highlights the need for participatory development of quality improvement indicators that are relevant at facility-level. Another national-level respondent elaborated on this:

‘You don't just send indicators—“these are the quality indicators.” You may have those universal kind of indicators, and those may be good at district, national level. But at the facility level, they need to develop those themselves, or be assisted to develop, so that they can monitor interventions that they have.’ (IV35-NS)

Such localization enables health facilities to address context-specific health challenges that might otherwise be overlooked. A HCW illustrated this point:

‘It helps to know what kinds of patients we're dealing with in our specific area. For example, here in our facility, we have many patients with hypertension. As a result, we see many cases of eclampsia. Someone might ask, “why do we keep seeing the same problems in patients from this area? What's happening there? Perhaps we should follow up.’ (IV20-HCW)

This underscores that quality improvement requires measurement systems that are flexible enough to be tailored to local disease burdens and priorities.

In short, routine maternal and neonatal health data do not fully capture the complex, multifaceted nature of quality of care. Standardized data lack sufficient detail and contextual richness to reflect critical dimensions of care quality, and the patient experience is notably absent. This creates an imbalance where data may meet the needs of stakeholders positioned outside the immediate care provision context, but do not capture the information frontline HCWs consider most relevant for meaningful quality improvement. When asked about why different stakeholders have divergent views on what constitutes useful data, one nurse-midwife explained:

‘Differences exist because of responsibilities. Because those who work in administration and those who work here are different—here it's a nurse, while in administration you find it's a health secretary or a doctor who isn't connected with things from here, or it's an officer who deals with statistical matters. You find differences, and sometimes it becomes difficult to understand, to have one goal or to think equally.’ (IV24-HCW)

This highlights that when different stakeholders’ goals and priorities diverge too strongly, the boundary object ‘web’ becomes overstretched. The metaphorical distance between highly contextualized clinical realities and higher-level data needs becomes too great for the RHIS to bridge and it cannot fulfil its boundary object function.

### Bigger picture: data extraction vs. local-level data use

The limitations identified—where routine data provide only a partial picture of quality and fail to bridge different stakeholder needs—are not simple, technical shortcomings but reflect structural issues. Particularly interviewees at national level extensively shared views on how deeply rooted patterns continue to influence contemporary health information practices in Tanzania. Several respondents characterized the RHIS as having been designed first and foremost to lift data ‘upward’ for use at higher administrative levels, rather than serving HCWs’ needs:

‘It’s based on this more extractive… I call it an extractive model, of reporting and extracting data away. […] It's a sort of authoritarian control function: ‘we want to know what you're up to.’ (IV44-NS)

This ‘extractive’ model has historical roots that can be traced back to pre-independence governance structures, when health information systems were initially established in Tanzania not with the primary purpose of supporting clinical care, but for surveillance and control. As another national-level respondent explained:

‘Even in the colonial era […] If you look at the design, it's more about collecting data and not treating the patient. It's knowing the aggregate data. It's about surveillance, control and the likes.’ (IV34-NS)

Elements of this historical legacy have persisted through subsequent iterations of the health information system. Interviewees described the design process as typically beginning from high-level priorities, with data collection requirements then cascading downward to health facilities:

‘We thought the best way to start would be from the apex. What is the goal you want to achieve? Where do you want to go, the targets? What indicator can you use for these targets? And then you break down the indicators. “Oh, so *you* need to collect data so that *we* can fill in this.”’ (IV37-NS)

Emphasizing the distinction between *you* and *we*, this stakeholder illustrates the hierarchical relationship where HCWs collect data so higher levels can complete required reporting.

Another systemic constraint relates to the fragmented nature of the Tanzanian RHIS landscape. This fragmentation, manifested in the existence of multiple partially overlapping systems and platforms (Table 2), is linked to the historically widespread vertical programming in the country’s health system. As one national-level stakeholder explained (IV44), ‘Tanzania comes from a vertical approach’ where ‘the whole health system has been built on programmes’ including reproductive health, immunization, malaria, TB, and HIV programs. This vertical structure became embedded in the health system's architecture: ‘that's the structure you see at the Ministry, and then the whole M&E [monitoring and evaluation] system has been crafted’ around these separate programs. Several interviewees discussed how Tanzania’s heavy financial dependence on external partners and international donors has been a major contributing factor to vertical programming in the health sector. As donors seek to measure the impact of their investments through parallel reporting structures, this has also perpetuated fragmentation in the health information system:

‘We are a partner-driven country in terms of investments […] So if I'm reporting to my donor on X service there, I’ll try to put investment so that the components will be captured and reported. Whether I introduce a parallel, a paper system, or I can employ data clerks who can collect and report it back.’ (IV36-NS)

These parallel systems contribute to a heavy documentation burden for HCWs. Rather than working with one system—a coherent boundary object that serves multiple purposes—care providers must navigate disconnected requirements which often duplicate efforts and fragment attention. A maternity ward in-charge (IV9) described how the same information must be recorded across multiple formats: ‘at the same time you fill here, at the same time you fill there’ across partographs, mother's cards, child's cards, and computer systems. This forces staff to divide their time between direct care and multiple documentation tasks, where ‘if you're two or three people, one takes care of the mother and child’ while others focus solely on filling different forms. The interviewee felt consolidation would be preferable: ‘If they just said: “now we don't want paper, we just want computer”—OK, let's put it all there, struggle with that one thing, I think it would be possible’ (IV9-HCW).

These systemic and structural constraints ultimately shape the perception that health data primarily serve external stakeholders, rather than the immediate interests of women, newborns, and the HCWs who care for them. Many of the interviewed HCWs did not view data as their core responsibility. As one nurse-midwife explained, there are widespread ‘negative perspectives about filling these records to be the responsibilities of other people and “my responsibilities are these”’ (IV12). Documentation duties were something many HCWs dreaded and often rushed through: ‘they just do things superficially without really putting their mind to it (*bila utulivu wa akili*—literally “without tranquillity of mind”)’ (IV12-HCW). These views of documentation cannot be seen in isolation from system design, which prioritizes higher-level data needs:

‘The system is not set up for them! […] They're doing it for someone else, and they're not even doing it for the patient. It's not for them and it's not for the patient.’ (IV44-NS)

Consequently, facility-level data use by care providers remains limited:

‘That’s not the habit in this country. […] We don’t have that data consumption, to utilize data at that level for the clinical management. So they collect, and “they will deal with it up there” (*watafanya wao huko juu*).’ (IV36-NS)

The mindset that data mostly serve those ‘up there’ reveals how the RHIS seems to operate as a hierarchy of extraction rather than a web of information exchange. This prevents routine maternal and neonatal health data from fulfilling their potential as a boundary object, bridging between clinical care and systemic quality improvement.

### Future picture: reimagining Tanzania’s RHIS

Having explored how structural constraints shape what routine health data can and cannot tell us about the quality of maternal and neonatal health services in Tanzania, we now turn to examine emerging initiatives that might offer pathways forward. Tanzania’s RHIS is currently in active transition, and the roll-out of GoTHOMIS presents a shift towards individual electronic health records. Many interviewees saw this as a promising evolution which offers opportunities for deeper understandings of quality of care. Individual health records provide a significantly more granular understanding of the quality-of-care patients receive compared to the aggregated data captured through MTUHA:

‘Process quality indicators are hard to capture, especially through the MTUHA system where you can't look at individual patient care. […] So you kind of get lost—I think what you need for that to work is individual health records.’ (IV44-NS)

HCWs pointed out how individual clinical records can also have a direct positive impact on quality of care. The current fragmentation of patient information creates clinical challenges, as explained by an intern doctor: ‘without incorporated case notes, it becomes a challenge because I don't know how she was managed when she delivered, how did they help this mother?’ (IV11-HCW). Care continuity can be threatened by fragmented clinical information, particularly in the case of obstetric complications. Individual patient records would address this challenge:

‘I would like to see that the system is uniform […] and specific for each patient. And access—that if I want previous notes, I get them. It will help a lot.’ (IV11-HCW)

By centering documentation of individual patient journeys in individual health records, the RHIS could grow into a genuine clinical resource that also supports quality improvement at multiple health system levels.

Such a development would require a fundamental shift in the purpose of Tanzania’s RHIS. One national-level respondent described it as a ‘paradigm shift’ in whose priorities inform system design:

‘We need a paradigm shift […] in terms of, OK, the development of solutions for data capture should be part and parcel of the patient care. That it’s not a tool that will be brought by the HMIS team or ICT team, no. It has to be a tool that doctors, nurses, pharmacists see as relevant and aligned to their workforce, and bringing something. That's where you can really get clinically relevant data and then the operational, financial, administrative, regulatory aspects will come in.’ (IV34-NS)

In this stakeholder’s view, the system should be built around clinical relevance first, with the overarching goal to improve care outcomes and experience for mothers and newborns. This would create a hierarchy where maternal and newborn wellbeing comes first, followed by supporting HCWs in delivering quality care, and finally meeting administrative monitoring needs. Such an approach requires participatory processes involving HCWs in system design processes:

‘If you want to collect data, don't ask the managers what data is to be collected. Ask a physician what data is to be collected.’ (IV37-NS)

Respondents were hopeful that a system designed with clinical utility as a primary purpose would also help promote internal motivation among HCWs, instead of mere compliance with external demands. This motivational aspect is key, as another respondent observed:

‘The health system should start with ‘what's the benefit of doing this?’ So that it's not felt as a burden.’ (IV43-NS)

Reorienting the RHIS to prioritize care over control would help to more effectively meet the data needs of people across health system level from the bottom-up.

Achieving more comprehensive measurement of quality of care also requires incorporating patient experiences. New approaches to capture experiences of care are currently in the works in Tanzania, representing another critical evolution in how quality of care is measured and understood. The *Afya yetu, huduma zetu* (Our health, our services) initiative stands out as a promising digital client feedback mechanism which can add a crucial dimension to measurement of quality of care. *Afya yetu, huduma zetu* was first developed as system specifically focused on maternal and child health care, but it is being expanded to extend to all health services. Clients provide feedback through their mobile phone or via community health workers, linking with the Unified Community Solution (UCS). A national-level stakeholder highlighted the potential of this system for providing a direct channel for patient voice:

‘It's an amazing wealth of data, but what we want is that this becomes routine. As soon as someone's visited a health facility, that they can provide feedback on the services they've received. So this is something where Tanzania is really a bit in the forefront.’ (IV44-NS)

Clients’ answers to questions are linked to DHIS2 and can be visualized in score cards for easier use across health system levels. By incorporating patient experiences into routine health information, Tanzania’s RHIS could bridge not just between clinical and administrative perspectives but also include the vital perspective of services users themselves, strengthening its boundary object function.

## Discussion

Why do we capture information about the care women and babies receive? What purpose and whose needs are we aiming to serve? These are questions that are not asked frequently enough. These are also questions that reveal complex tensions at the heart of RHIS—between external accountability and local ownership, between standardized reporting and contextual relevance, and between reported numbers and true change. Questions about purpose and information needs are particularly salient when it comes to measuring quality of maternal and neonatal care. Our qualitative findings highlight how in Tanzania’s RHIS, routine maternal and neonatal health data are used differently by different stakeholders. Boundary object theory provides a useful lens for understanding how health information systems ideally provide a bridging function. Returning to our web metaphor (Fig. 1), the RHIS creates connecting strands that allow for collaboration between diverse stakeholders through shared information, while being flexible enough to accommodate specific information needs (Star and Griesemer 1989, Star 2010). We identified examples of this potential, where routine health data can provide a shared vocabulary to communicate about quality of care. Information relevant to clinical quality of care can, once aggregated, serve planning and monitoring needs at higher system levels. However, we also found examples where the boundary object function breaks down. Our interlocutors across health system levels consistently acknowledged that although the RHIS can give us a sense of the structural context of care delivery and of health outcomes, much of what matters for care processes—including both the provision and experience of care—remains uncaptured. There is limited availability of fine-grained, context-specific information about quality of care.

Considering the complex, multidimensional nature of quality of care, it is inevitable that quantitative routine health data will only provide a partial picture of the care women and newborns receive. Given that different people have different perspectives on what constitutes quality, capturing all dimensions of care quality simultaneously is not feasible, nor is it necessary. If purposeful, partial pictures can still be useful. However, the type of partial picture that is useful depends on a person’s role in supporting quality of care. Some dimensions of care quality can be standardized across contexts, and for these, a core set of indicators shared by all stakeholders enables tracking and comparison across sites and health system levels (WHO 2025). Yet, our findings highlight that meaningful quality improvement also requires flexible and adaptable measurement. For a quality improvement team at facility level, for example, it makes sense to monitor detailed process indicators for specific improvement strategies they are implementing, while district managers should track fewer process indicators across multiple sites and focus on management functions like resource allocation. Regional and national policymakers need an even smaller set of high-level indicators to track progress for broader health system goals (WHO 2025). This differentiation aligns with boundary object theory: success depends not on standardization, but on flexibility that allows different groups to make information more specific and tailored to their local context and needs (Star 2010).

In thinking about the purpose and number of quality-of-care data elements at each health system level, a pyramid structure offers a useful visual framework. At the bottom of the pyramid—the community and health facility levels—health data should be most comprehensive, with progressively fewer indicators monitored at higher system levels (AbouZahr and Boerma 2005). This approach prioritizes information needs in the places where care is sought and delivered, subsequently distilling key insights for higher-level planning. This is not the order of priorities currently shaping Tanzania's RHIS, where design is largely driven by standardization needs for higher-level oversight (Unkels et al. 2023). Indeed, we observed tension around stakeholders’ varying views on what is the main purpose of routine health data. Interviewees across health system levels agreed that quality-of-care measurement is important but had divergent perspectives on whose needs it should meet and how. This echoes qualitative findings from Ethiopia showing ‘limited clarity’ about whether measurement of health care quality should primarily serve individual patient care or system performance tracking (Lemma et al. 2024).

It is not a question of either/or—systems can be designed to capture information that serves both clinical care and higher-level purposes. Nonetheless, it is crucial to shift focus towards collecting data that are useful to the people who are tasked to document it at the point of care. Without this shift, data quality will remain problematic. Studies across LMICs describe how low perceived relevance and feasibility dampen motivation to collect and report data among HCWs, which negatively impacts data quality and reinforces low perceived usefulness of data in a negative feedback loop (Molenaar et al. 2024a). Fragmentation will also persist, as HCWs continue to improvise parallel documentation systems to fit their own data needs (Unkels et al. 2023). As noted by Mahundi et al (2018), there are many locally improvised tools in Tanzania which are ‘meant to either cover a certain deficiency or correct a certain misunderstanding’, particularly in the domain of maternal and neonatal health services.

Shifting priorities in RHIS is a fundamentally political process, involving contested questions of power, control, and whose knowledge counts in system design. We have shown how ‘extractive’ data systems that depart from high-level priorities have formed under the influence of historical and structural drivers. Multiple stakeholders with varied interests—government, donors, implementing partners—shape RHIS design in Tanzania, often prioritizing top-down accountability over local usefulness (Nyella and Kimaro 2015). This extractive approach has also been noted in externally-funded periodic surveys that are ‘rarely directly fed back into the improvement of routine services’ (Agweyu et al. 2023). In contrast to retrospective surveys, RHIS offer unique potential to provide actionable, near real-time information for health system improvement, but only if they avoid replicating these same extractive patterns.

Digitalization may help with this. Digital tools and platforms alone cannot address the systemic barriers to recording accurate, timely information about women and newborns (Molenaar et al. 2024b), and we must avoid adopting solutionist or techno-utopian perspectives on RHIS digitalization (Greenhalgh et al. 2009). However, electronic health records and digital patient feedback systems such as *Afya yetu, huduma zetu* present opportunities to capture more fine-grained information that is valuable for quality improvement efforts. Realizing this potential requires a pragmatic approach. Measurement approaches must remain feasible and avoid system overload, with recent WHO guidance advising to ‘start where you are, with the resources you have’ and emphasizing the need to balance ‘ideal’ versus ‘good enough’ quality of care indicators (WHO 2025). We must ensure that time spent on data capture is proportional to its use and prevent digitalization from creating ‘too much data too soon’ (Shamba et al. 2021). Finally, organizational readiness is essential, as technical solutions alone are insufficient without the leadership and change management capabilities needed to translate data into meaningful improvement in care (Agweyu et al. 2023).

## Study strengths and limitations

We consider the combination of observations and semi-structured interviews across Tanzanian health system levels to be a key strength of this study. These qualitative methods allowed for an in-depth exploration of relevant behaviours, perceptions and non-articulated dynamics that might otherwise remain hidden. Methodological triangulation, combined with immersion in relevant literature and policy documents, strengthened our understanding of both formal and informal data practices. Boundary object theory offered a useful analytical lens to explore how Tanzania’s RHIS succeeds in bridging stakeholder needs in quality-of-care measurement and where its limits lie. It helped connect current RHIS characteristics to deeper historical and organizational patterns and enabled critical reflections on key considerations in future system (re)design. This study was geographically focused on Tanzania, which limits the generalizability of findings to other country contexts with different health systems and historical trajectories. Facility-level findings were from two hospitals in Mtwara region and therefore do not represent perspectives from lower-level providers of childbirth care and from other Tanzanian regions. Additionally, the data collection took place during a period of active transition for Tanzania’s RHIS—notably the rollout of GoTHOMIS and shift towards individual patient records—and stakeholders’ experiences and perspectives are therefore likely to be in flux, affecting the relevance of our findings over time. We recommend that future research should also explore service users’ perspectives on how their experience of care can be best incorporated in Tanzania’s RHIS.

## Conclusion

Routine health data provide a valuable but partial picture of the quality of maternal and neonatal care in Tanzania. Quality of care is inherently complex and multifaceted, making it challenging for any single information system to bridge varying interpretations and priorities. Rigid standardization of quality-of-care measurement is untenable and will fail to accommodate local quality improvement needs. A more flexible and adaptable approach is needed, designed around differing information needs from the bottom-up. Achieving this requires a shift in current RHIS priorities. The emphasis on external oversight rather than local-level data use has led to the widespread perception that data only serves ‘those up there’ and prevents the RHIS from functioning effectively as a boundary object. Our findings highlight how health information systems work best when they create value for those who generate the data, not just those who consume it for oversight purposes. This requires reorienting systems to prioritize care over control, to better serve information needs across all health system levels. Individual electronic health records hold promise to improve Tanzania’s RHIS, particularly when combined with patient feedback mechanisms that could bridge clinical, administrative, and service user perspectives. However, transforming RHIS ultimately requires more than technical solutions—it demands fundamental shifts in who defines measurement priorities and whose knowledge counts in system design.

## Supplementary Material

czag030_Supplementary_Data

## Data Availability

As participants were promised confidentiality, we are unable to make our data publicly available. However, excerpts of field notes and interview transcripts (carefully screened for potentially identifying details) can be made available upon reasonable request to researchers who complete a data sharing agreement.

## Reflexivity statement

Our research team included members from both high-income and low-middle income countries, with good gender balance (four women and three men). J.M., the first author, is a PhD candidate with Dutch nationality based in Belgium, supervised by L.B., J.V.O., C.H., and A.B.P. The primary affiliations of J.M., L.B., and J.V.O. are with institutions in Belgium, while A.K., Z.J., and A.B.P. are primarily affiliated with institutions in Tanzania. C.H.’s primary affiliation is with a Swedish institution. The researchers’ background and positions affected the angle of investigation that was chosen; the methods that were selected; the findings that were considered relevant to publish; and how they were framed in the final manuscript. As a non-Tanzanian woman without clinical training, J.M. was considered an outsider by study participants. For the ethnographic observations, her positionality played out differently in the two hospitals: at hospital A, labour ward staff were used to having visiting international students, while at hospital B, foreign visitors were rare and the concept of observation was less familiar. Being accompanied by Tanzanian colleagues for initial introductions helped ensure that objectives were clearly communicated, making it clear that researchers by no means aimed to identify ‘incorrect’ reporting practices nor blame specific individuals or institutions. Not being a medical professional made it easier to justify why she stayed strictly out of clinical tasks. Being of a similar age to many of the nurse-midwives seemed to help build rapport and reduce power differentials. For interviews with health sector managers and policymakers at district, regional and national-levels, power dynamics were different. With more senior respondents, interviewers (J.M., Z.J., and A.K.) were relatively junior in terms of both age and professional standing. This positioning likely influenced the types of responses we received—some senior managers may have felt compelled to present official narratives about RHIS effectiveness. Conversely, our status as external researchers without direct authority over health system operations may have encouraged some participants to speak more openly about challenges and workarounds that they might not discuss with supervisors or funders. J.M. developed the overall study protocol and topic guides and refined them based on input from the other team members. Daily debrief meetings between data collectors, as well as online discussions with team members, informed preliminary analyses and further data collection through topic guide adjustments and additions. J.M. speaks intermediate-level Swahili, and her knowledge was good enough to carry out coding on original transcripts. In cases of ambiguity, native Swahili speakers on the team (especially A.K.) helped clarify. The choice to code Swahili transcripts was carefully considered—we felt that translating all transcripts to English prior to coding would bring significant risk of nuances being lost, especially if done by an external translator without in-depth knowledge of the subject matter. Manuscript writing was led by J.M., beginning with a paper outline that coauthors provided input on. After J.M. prepared a full draft, all coauthors gave written feedback. All coauthors had sufficient time and opportunity to provide input and review the manuscript, and potential power asymmetries among junior and senior researchers were navigated through one-to-one communication, orally or through informal channels such as WhatsApp.
