# Supplementary material for: Partial pictures: what routine health data can and cannot tell us about the quality of maternal and neonatal health services in Tanzania
Source: Health Policy Plan. 2026 Mar 5;41(5):747–60. doi: 10.1093/heapol/czag030 (PMC13187646; doi:10.1093/heapol/czag030)
Supplement: czag030_Supplementary_Data [file czag030_supplementary_data.docx]

**Topic guides**

These topic guides were used for the qualitative interviews conducted with different stakeholders during two data collection periods in 2023 and 2025. Due to the iterative nature of qualitative research, the topic guides were adjusted over time to better fit the evolving nature of the research objectives and the insights gained in earlier stages of the research. Additional questions and edits were included directly in the Swahili versions while data collection was ongoing, but we made efforts to translate and include these in the English versions here. Please note that not all minor tweaks and variations are represented in the versions in this document.

The interviews were semi-structured, meaning the order of the questions was not fixed. The interviewers attempted to follow the flow of the participant’s narrative as much as possible. Most of the probes included in the guides were typically not actually asked, as interviewees would have already covered this in their responses to previous, more general questions. Language and phrasing were adapted to specific participants. Questions were also adjusted to match the expertise and professional position of the interviewees.

The focus of the topic guides used in 2023 and 2025 differed. In the 2023 data collection in Mtwara, we focused mainly on 1) general experiences with routine maternal and neonatal health data and 2) specific experiences with a perinatal eRegistry implemented in the context of the ALERT project. In 2025 interviews, we focused on exploring key stakeholders’ views of how Tanzania’s data systems for maternal and neonatal health have evolved over time, how the information is used for decision-making, and quality-of-care measurement.

Table of Contents

[2023 Topic guide for labour ward HCWs 2](#_Toc207008318)

[2023 Topic guide for managerial/administrative stakeholders 5](#_Toc207008319)

[2025 Topic guide for key informants in Tanzania at regional and national level 8](#_Toc207008320)

## **2023 Topic guide for labour ward HCWs**

**Briefing & introduction:**

Thank you for agreeing to be interviewed. Before starting, I will give you an information sheet to read and a consent form to sign. Please take all the time you need to read through this and ask any questions you might have. I will ask you a series of open-ended questions to learn about your personal experiences and opinions. There are no right or wrong answers, and if there are any questions you prefer not to answer please let me know, and then we can move onto another question. The interview will take about 1 hour of your time. This is not an evaluation or a test: we value your opinions and your help is important.

**Background**

1. Can you please introduce yourself and tell me about your position here at the hospital?
2. Can you tell me about the ALERT activities you have been part of?

**General aspects of data collection and recording**

*Focus: to explore general experiences of data collection and reporting, as well as key barriers and facilitators.*

1. I have noticed there are many different forms and registers where data is recorded about women and their babies. I am curious about your experiences with these and the different challenges they have. Can you tell me about the different forms and registers that are used on the ward? (can discuss each one separately)

- Can you tell me about your daily routine of using these?
- At what moment do you do this task? How much time does it take?

1. Can you tell me about things or factors that help you completing your documentation duties?
2. Can you tell me about things or factors that hinder you (make it challenging for you) to complete this task?
3. Can you explain how the responsibilities for documentation and reporting of data are divided?

- In your experience, is it always clear who is responsible?
- In your view, is the work fairly divided?
- How do you and your colleagues work together to complete documentation and reporting tasks?

**Quality (completeness, accuracy, timeliness)**

*Focus: firstly explore the participant’s own definition of ‘good’/quality data, secondly explore different aspects of data quality (completeness, accuracy, timeliness) more specifically.*

1. People often talk about the **quality** of data, but not everyone defines ‘quality’ in the same way. From your point of view, what makes data collected about mothers and babies of good quality?
   - In an ideal case scenario, what are all the characteristics data should have to be of good quality?
   - Do you think quality of data might be understood differently by other people?
     - For example: Do you think hospital leaders have the same view as you of what makes data of high quality? Do you think officials at the district and regional level have the same view of what makes data of high quality?
2. Please, let us think about the **completeness** of data (the extent to which all patients are included, and all data variables are recorded). Can you think of reasons why data might be incomplete sometimes?

- For example, what might be reasons that information about a particular woman is not recorded in the paper register? For example, if there is an obstetric complication – what might be reasons that this is not recorded in the register?

1. Next, please let us think about the **accuracy** of data (if the information is correct and reflects exactly what happened). Can you think of issues that influence the accuracy of the data?

- For example, what might be reasons that information recorded on a partograph does not reflect exactly what care a woman actually received?

1. Next, please let us think about the **timeliness** of data (whether it is available and shared when asked/required). Can you think of reasons why there might be delays in reporting of data?

- Can you think of an example from your personal experience when there was an issue with the timeliness of data? What were the reasons for the delay?

**Limits of the data**

1. Thinking about the routine data collection on the ward, is there any information you feel is important that is **not** captured in the data?

- Are there things which are important for good quality care for women or their babies, which are not reflected in the data?
- If a person just looks at the data, what are things they might not understand about the services being provided?

**Data use**

1. Can you tell me about how the data you help collect and report are used?

- Where do the data go, after they have been collected at the hospital?
- Do you get feedback on the data?
- What is this feedback like?
- How is this (feedback) system working for you?
- Are the data used to inform changes or decisions in your hospital?

1. Can you share any experiences of a time when you yourself, or your team, used data?

- How did you go about making sense of the data? What information did the data give you, and how did you act upon it?

1. In an ideal scenario, how do you think data about women and babies should be used? How can the data inform patient care and your work?

**Perinatal eRegistry**

1. At the moment there is the perinatal eRegistry at your hospital. Can you tell me about your initial personal experience when you first heard about the perinatal eRegistry?

- What did you think when you first learned about the eRegistry?
- Were there things that surprised you about the perinatal eRegistry?
- Were there specific things you struggled with or were confused about in the beginning?

1. From your personal experience, did the perinatal eRegistry change how information about mothers and babies is reported at your hospital?

- Which changes do you like? Which changes do you dislike?
- Are there any differences in the ways data is used now that the eRegistry is there, compared to before?

1. Can you think of reasons why there might be differences between the data in the paper registers and the eRegistry?
2. From your experience, how would you describe the quality of the data reported in the eRegistry?

- What are the main strengths?
- What are the main shortcomings?
- What are the challenges which make it hard to record data of high quality? How could quality be improved?

1. From your perspective, would you like to have an electronic register at your hospital in the long run?

- Why (not)? What are the most important (dis)advantages?

1. Do you think it would be possible to keep the eRegistry running in the long term in this hospital?

- Why (not)?
- What are the main barriers to keeping the eRegistry running?
- How could these barriers be addressed?

**Wrapping up & debriefing**

Do you have anything else you think would be relevant to mention or to add?

Thank you very much for taking the time for this interview. If you would like to stay informed about my project and my findings, I can note down your contact details and I will be in touch again.

## **2023 Topic guide for managerial/administrative stakeholders**

**Briefing & introduction:**

Thank you for agreeing to be interviewed. Before starting, I will give you an information sheet to read and a consent form to sign. Please take all the time you need to read through this and ask any questions you might have. I will ask you a series of open-ended questions to learn about your personal experiences and opinions. There are no right or wrong answers, and if there are any questions you prefer not to answer please let me know, and then we can move onto another question. The interview will take about 1 hour of your time. This is not an evaluation or a test: we value your opinions and your help is important.

**Background**

1. Can you please introduce yourself and tell me about your position?
2. Can you tell me about the ALERT activities you have been part of?

**General aspects of data collection and recording**

*Focus: to explore general experiences of routine data collection and reporting about women and babies, as well as key barriers and facilitators (adjust phrasing for higher-level stakeholders).*

1. I have noticed there are many different forms and registers where data is recorded about women and their babies. I am curious about their purpose, their use and the different challenges they have. Can you tell me about the different forms and registers that are used at this hospital? (can discuss each one separately)

- For whom (on whose behalf) are these data collected?

1. Can you explain how the responsibilities for documentation and reporting of data are divided the maternity ward at your hospital?

- In your experience, is it always clear who is responsible?
- In your view, is the work fairly divided?

1. Can you tell me about the challenges maternity care providers face in completing their documentation and reporting tasks? Can you tell me about things or factors that help care providers to complete their documentation and reporting tasks?
2. Can you tell me about the way you supervise and support care providers to complete their documentation duties?

**Quality (completeness, accuracy, timeliness)**

*Focus: firstly explore the participant’s own definition of ‘good’/quality data, secondly explore different aspects of data quality (completeness, accuracy, timeliness) more specifically.*

1. People often talk about the **quality** of data, but not everyone defines ‘quality’ in the same way. From your point of view, what makes data collected about mothers and babies of good quality?
   - In an ideal case scenario, what are all the characteristics data should have to be of good quality?
   - Do you think quality of data might be understood differently by other people? For example: Do you think health workers have the same view as you of what makes data of high quality?
   - Do you think officials at the district and regional level have the same view of what makes data of high quality?
2. Please, let us think about the **completeness** of data (the extent to which all patients are included, and all data variables are recorded). Can you think of reasons why data might be incomplete sometimes?

- For example, what might be reasons that information about a particular woman is not recorded in the paper register? For example, if there is an obstetric complication – what might be reasons that this is not recorded in the register?

1. Next, please let us think about the **accuracy** of data (if the information is correct and reflects exactly what happened). Can you think of issues that influence the accuracy of the data?

- For example, what might be reasons that information recorded on a partograph does not reflect exactly what care a woman actually received?

1. Next, please let us think about the **timeliness** of data (whether it is available and shared when asked/required). Can you think of reasons why there might be delays in reporting of data?

Can you think of an example from your personal experience when there was an issue with the timeliness of data? What were the reasons for the delay?

**Limits of the data**

1. Thinking about the routine data collection on the labour ward of your hospital (not just the eRegistry), is there any information you feel is important that is **not** captured in the data?

- Are there things which are important for good quality care for women or their babies, which are not reflected in the data?
- If a person just looks at the data, what are things they might not understand about the services being provided?

**Data use**

1. Can you tell me about how the data collected about women and babies are used?

- Where do the data go, after they have been collected at the hospital?
- Do you get feedback on the data?
- What is this feedback like?
- How is this (feedback) system working for you?

1. Are the data used to inform changes or decisions in your hospital?

- Can you share any experiences of a time when you yourself, or your team, used data?
- How did you go about making sense of the data? What information did the data give you, and how did you act upon it?
  - In an ideal scenario, how do you think data about women and babies should be used? How can the data inform patient care and your work?

**Perinatal eRegistry**

1. At the moment there is the perinatal eRegistry at your hospital. Can you tell me about your initial personal experience when you first heard about the perinatal eRegistry?

- What did you think when you first learned about the eRegistry?
- Were there things that surprised you about the perinatal eRegistry?
- Were there specific things you struggled with or were confused about in the beginning?

1. From your personal experience, did the perinatal eRegistry change how information about mothers and babies is reported at your hospital?

- Which changes do you like? Which changes do you dislike?
- What do you see as the benefits of the eRegistry for your work?

1. Are there any differences in the ways data is used now that the eRegistry is there, compared to before?
2. Can you think of reasons why there might be differences between the data in the paper registers and the eRegistry?
3. From your experience, how would you describe the quality of the data reported in the eRegistry?

- What are the main strengths?
- What are the main shortcomings?
- What are the challenges which make it hard to record data of high quality? How could quality be improved?

1. From your perspective, would you like to have an electronic register at your hospital in the long run?

- Why (not)? What are the most important (dis)advantages?

1. Do you think it would be possible to keep the eRegistry running in the long term in this hospital?
   - Why (not)?
   - What are the main barriers to keeping the eRegistry running?
   - How could these barriers be addressed?

**Wrapping up & debriefing**

Do you have anything else you think would be relevant to mention or to add?

Thank you very much for taking the time for this interview. If you would like to stay informed about my project and my findings, I can note down your contact details and I will be in touch again.

## **2025 Topic guide for key informants in Tanzania at regional and national level**

**Briefing & introduction:**

- Thank you for agreeing to be interviewed.
- Before starting, I will give you an information sheet to read and a consent form to sign. Please take all the time you need to read through this and ask any questions you might have.
- I will ask you a series of open-ended questions to learn about your experiences and opinions. There are no right or wrong answers, and if there are any questions you prefer not to answer please let me know, and then we can move onto another question. The interview will take about one hour of your time.
- This interview aims to understand your experiences and perspectives on maternal and newborn health data systems in Tanzania, including both routine health information and periodic assessments. We're particularly interested in how these data systems have evolved over time, how the information is used for decision-making, and your thoughts on measuring and improving quality of care. The discussion will also touch on newer initiatives like the Kanzi data initiative which is used in some regions, and your views on future developments in this field.
- Before we really get started with the interview, I would like to ask some basic questions about your training and professional experience.

**Background & starting**

1. Can you tell me about your role and your main responsibilities?
   - Current job title
   - Training/educational background (cadre)
   - Total years of professional experience
   - Years in current role

**Historical context & changes over time**

1. How do you view the current situation regarding maternal and neonatal health in Tanzania?
   - How do you think international maternal and neonatal health goals (MDGs, SDGs) influenced Tanzania's approach to these issues?
2. Can you describe the changes you have seen in how maternal and newborn health data is collected and used in Tanzania?
   - Which of these changes do you consider most significant and why?
3. What types of maternal and neonatal health data do you use in your work?

**Different data sources: routine health data & periodic surveys/assessments**

1. Are there any changes you know of in how routine maternal and newborn health data is collected at health facility level? (Please specify.)
   - What drove these changes?
   - How has the introduction of digital health information systems affected the quality and use of these routine data?
2. Which periodic surveys and assessments do you think have been most influential in shaping maternal health policy in Tanzania? Why?
3. How do you see the relationship between routine data systems and periodic assessments evolving?
   - Are these different data sources becoming more integrated/aligned or remaining distinct?

**Data use and decision-making**

1. Could you give me an example of an MNH indicator which you consider particularly *useful* in your work?
   - What is this indicator useful for? What are some examples of how this indicator has informed decision-making or policy changes?
   - Do you think this indicator is also useful for people working at different (lower/higher) levels of the health system? Why (not)?
2. Can you give some examples of a routine indicator that you consider less useful?
   - E.g. an indicator that is hard to interpret and act upon?
   - E.g. an indicator that you have problems getting accurate and timely data for?
3. Could you share an example of how routine maternal and neonatal health data has directly influenced policy or practice in your work?
4. What would help make routine health data more useful for decision-making at different levels of the health system?

**Accountability: responding to data trends (regional stakeholders ONLY)**

You have already talked about how maternal and neonatal health data can inform policy and decision-making. I'd like to understand how at a more local level, maternal and newborn health data are used to monitor service delivery and drive improvements [in the region]. Let me share some specific situations.

1. Imagine during a monthly review you discover that in the past six months, one facility had much lower ANC attendance than its usual attendance rates. What would be the immediate and longer-term responses?
2. Imagine a situation where a facility consistently outperforms others in terms of neonatal health outcomes. How might this information be used to improve practices elsewhere?
3. If during data verification you find that birth weights weren't recorded for 40% of newborns in a facility last month, what would be the process for addressing this?

**Quality of care measurement**

I would like to know your views on various aspects of quality of care that should be considered in measuring maternal and newborn health services. (Optional additional intro: While we've made progress in measuring service coverage and basic health outcomes, understanding and capturing the quality of care remains complex. This can include both the *clinical* aspects of care - like following protocols and standards - and the *experience* of care from the perspective of mothers and families. I'm particularly interested in your views on how well our measurement approaches can capture these different dimensions of quality.)

1. How can we use data to understand the quality of care women and newborns receive in health facilities?
   - How do current systems monitor clinical quality of healthcare?
   - How do they monitor how patients experience and feel about the quality of care from their own perspective? (experience of care, person-centered care)
2. In your experience, what are the challenges in collecting meaningful facility-based health data about quality of care at the health facility level?
3. How can health workers and supervisors balance recording quality of care with their other duties?
4. In what ways do community expectations about quality of care differ from what our formal measurement systems consider?

**Kanzi data**

In our research in Mtwara region, we observed the implementation of the Kanzi data system, which uses WhatsApp to report information about progress of labour of individual women.

1. Have you heard about this initiative? (Questions below ONLY if interviewee is familiar with Kanzi data)
2. Can you share your understanding of the Kanzi data system and its role in monitoring maternal health?
3. What kind of support or feedback do healthcare workers typically receive through the WhatsApp groups?
4. Can you describe a time when rapid information sharing across facilities made a difference in a maternal or neonatal case?
5. Have you encountered any challenges while using the Kanzi data system?
6. How does the time needed for Kanzi data reporting compare with other documentation requirements?
7. Some healthcare workers have mentioned feeling stretched between patient care and reporting requirements. What are your thoughts on this balance?
8. How does this type of monitoring influence workplace dynamics and relationships?
9. How do you balance the need for quick information sharing with patient privacy concerns?
10. If you could modify how Kanzi data reporting works, what would you suggest?

**Looking ahead & closing**

1. Looking ahead 5-10 years, what changes would you most like to see in how we measure and improve maternal and newborn care in Tanzania?
2. How do you think we can reduce the reporting burden on health workers without losing important information?
3. Can you suggest names of other actors who might be relevant to contact for this study? [If yes] Would it be okay if I used your name when I contact these people to say that you recommended them to me?

Thank you very much for taking the time for this interview. If you would like to stay informed about this project and its findings, let me know and I will be in touch again.
